# Combining dynamic generalized linear models and mechanistic modelling to optimize treatment strategies against bovine respiratory disease

**DOI:** 10.1186/s13567-025-01611-y

**Published:** 2025-09-25

**Authors:** Carolina Merca, Baptiste Sorin-Dupont, Anders Ringgaard Kristensen, Sébastien Picault, Sébastien Assié, Pauline Ezanno

**Affiliations:** 1 Department of Veterinary and Animal Sciences, Faculty of Health and Medical Sciences, University of Copenhagen, Grønnegårdsvej 2, 1870 Frederiksberg C, Denmark; 2 Oniris, INRAE, BIOEPAR, 44300 Nantes, France

**Keywords:** Decision support tool, early warnings, state‒space models, livestock health, epidemiology

## Abstract

Bovine respiratory disease (BRD) is a major health challenge for young bulls. To minimize economic losses, collective treatments have been widely adopted. Nevertheless, performing collective treatments involves a trade-off between BRD cumulative incidence and severity, and antimicrobial usage (AMU). Therefore, we propose a proof-of-concept of a decision support tool aimed at helping farmers and veterinarians make informed decisions about the appropriate timing for performing collective treatment for BRD. The proposed framework integrates a mechanistic stochastic simulation engine for modelling the spread of a BRD pathogen (*Mannheimia haemolytica*) and a hierarchical multivariate binomial dynamic generalized linear model (DGLM), which provides early warnings based on infection risk estimates. Using synthetic data, we studied 48 scenarios, involving two batch sizes (small and large), four farm risk levels for developing BRD (low, medium, balanced, and high), two batch allocation systems (sorted by risk level or randomly allocated), and three treatment interventions (individual, conventional collective, and DGLM-based collective). In high- and medium-risk scenarios, collective treatments triggered by the DGLM were associated with a reduction in BRD cumulative incidence and disease severity, especially in large populations. Compared with conventional treatments, DGLM-based collective treatments typically result in either lower or equivalent AMU, with the largest reductions being observed in medium-, balanced-, and high-risk scenarios. Additionally, the DGLM estimates of infection risk aligned well with the empirical risk estimates during the first time steps of the simulation. These findings highlight the potential of the proposed decision support tool in providing valuable guidance for improving animal welfare and AMU. Further validation through real-world data collected from on-farm situations is necessary.

**Supplementary Information:**

The online version contains supplementary material available at 10.1186/s13567-025-01611-y.

## Introduction

Bovine respiratory disease (BRD) is a prevalent and complex respiratory condition that affects cattle, particularly calves and feedlot cattle. The disease is characterized by inflammation of the upper and lower respiratory tracts, leading to coughing, nasal discharge, rapid breathing, fever, depression, and reduced appetite. It is caused by a combination of viral (e.g., the bovine respiratory syncytial virus) and bacterial pathogens (e.g., *Mannheimia haemolytica*). BRD can be triggered by various stressors that animals are commonly exposed to, such as transportation, commingling, adverse weather conditions, or poor ventilation [1]. BRD leads to reduced feed efficiency, growth rates, and overall productivity, resulting in substantial financial losses for producers [2, 3]. Additionally, the clinical signs linked to the disease cause significant suffering in affected animals. The prevention and management of BRD are crucial for maintaining animal health, welfare, and sustainable livestock production.

The use of antimicrobials (AMU) is the main strategy employed worldwide to control BRD in the cattle industry [4]. Animals can be treated individually upon appraisal of clinical signs, but collective treatments are also a common practice. The latter is a metaphylactic approach that consists of administering a dose of antimicrobial agent to an entire group of animals, while only part of the group is diagnosed with the disease to prevent additional cases [5]. The relevant threshold for such a collective intervention is, however, still largely questioned [6].

The concerns raised by antimicrobial resistance motivate the need for responsible treatment strategies. On the one hand, curative and metaphylactic treatments can reduce the occurrence of respiratory diseases [4, 7]. On the other hand, the overuse of antimicrobials promotes the positive selection of resistant bacterial strands, which can lead to their proliferation and the loss of efficacy of treatments [8], in both animals and humans. This trade-off highlights the need for targeted collective treatments and/or better usage of vaccination. Veterinarians and animal scientists also highlight the need to implement early warning systems and preventive measures [9].

State-space models have emerged as promising approaches for obtaining early warnings in the context of animal production health [10, 11]. These models often utilize a Bayesian framework to generate their estimates, incorporating both observed data and any prior knowledge that may be available prior to a given observation [12]. One key advantage of these models is their capacity to estimate the true state of an unobservable variable at each point in time [13]. These specifications make state-space models valuable tools for monitoring animal production, allowing the detection of potential issues early on.

Coupling state‒space models with mechanistic modelling could allow us to assess the impact of early warnings on the system. Mechanistic modelling is often used to simulate complex infection dynamics to comprehend and anticipate them. Such models can thus produce synthetic data, compare scenarios and rank interventions [14].

In the case of BRD, only two mechanistic models that rank treatment practices at the batch level have been published [15, 16]. The first simulates the circulation of an undefined pathogen with characteristics mainly based on observable clinical signs in conditions mimicking contrasted farming contexts such as small versus large batches (considered as isolated from other batches). Moreover, the batches could display contrasting risks (high vs. low) of BRD occurrence. The model featured a risk status defining the individual risk level of being infected and of shedding respiratory pathogens. This qualitative information (low, medium, or high) summarizes the level of stressors encountered by the individual prior to its arrival at the farm [17, 18]. The study highlighted greater BRD circulation in large high-risk batches, which could benefit the most from collective treatments. The second model developed within the team compares the impact of farm management practices on the spread of three causal pathogens of BRD on multibatch farms featuring several individual risk levels of contracting BRD. This study highlighted the need for prior knowledge on the individual risk of the animals to contract BRD, as grouping animals together in batches in accordance with their individual risk level of developing BRD limits the spread of the most contagious pathogens. While mechanistic approaches are relevant for assessing the value of early warning tools to make early and informed on-farm interventions, to the best of our knowledge, no coupling between such mechanistic models and automated early warning systems has been studied in the context of animal health.

Our objective was to establish a proof of concept for a decision support tool that combines automated early warnings and a mechanistic epidemiological model. The goal of this model was to identify when triggering collective treatments is relevant for BRD. Moreover, the model aimed at determining when individual treatments should be preferred to limit both AMU and BRD cases. Comparing contrasted  situations, we evaluated tool effectiveness in reducing the number of BRD cases, the duration of severe clinical signs and AMU. To that end, we used a pipeline that combines a mechanistic stochastic simulation engine modelling the spread of a BRD pathogen (*M. haemolytica*) and a hierarchical multivariate binomial dynamic generalized linear model, a type of state‒space model, applied to synthetic data. This tool enables proactive decision-making by initiating timely collective treatment interventions to minimize the spread and severity of the disease. By leveraging collective treatment strategies based on these early warnings, this system has the potential to increase the efficiency and effectiveness of BRD control programs.

## Materials and methods

### Processes and assumptions of the mechanistic model

We built our proof of concept on a pipeline modelling the spread of *M. haemolytica*. This pathogen is commonly reported in BRD outbreaks. Furthermore, the dynamics of an *M. haemolytica* outbreak have already been modelled within the team and have the advantages of limited stochasticity and repeatable behaviour [15].

The mechanistic part of the modelling pipeline was developed via the EMULSION framework [19]. This framework uses a Python simulation engine on models written as structured text files. The model was structured into processes described as finite state machines to describe states and transitions. This framework also allows the user to implement code add-ons written in Python to incorporate events with outputs coming from outside of the model (e.g*.,* data input).

We extended a previously existing model [15]. The model was individual-based and was written in discrete time with time steps of half a day. It simulated the first 40 days of fattening, i.e., the most at-risk period of BRD occurrence on fattening farms. It features a risk status defining the individual risk level of being infected and of shedding respiratory pathogens. The higher the risk is, the higher the infection rate and the more likely the animals are to shed pathogens if infected. The model monitored five processes: hyperthermia (either unspecific or due to BRD), health status (susceptible, infected, resistant), clinical signs (asymptomatic, or with mild or severe clinical signs), detection (detected or not), and treatment (treated or not). Hereafter, we detail how treatments were triggered in this new version of the model. Details on other (unchanged) processes and transitions can be found in the first section of the Supporting Information.

As in the previous model, any detected animal could be individually treated with antibiotics. A treatment dose had a success probability $pT$. In the case of treatment failure, the treatment could be repeated up to a maximum number of doses per animal ($maxT$). After the treatment period, the animals could transition back from treated to not treated, even if they still exhibited clinical signs after $maxT$ treatments.

In addition to individual treatment upon detection, collective treatments were included in the new model. Two criteria for collective treatment were implemented. First, conventional collective treatment could be performed once the cumulative number of detected animals reached a fixed threshold. In European fattening operations, a threshold of approximately 10% of the batch size is commonly used, although this value has not been backed by dedicated field or experimental observations since its original recommendation in 1977 [20]. Second, a collective treatment could also be triggered by automated alarms generated by the dynamic generalized linear model connected to the mechanistic model, as described in  the "Global Pipeline workflow" section.

### Scenarios

The analysis was conducted on 48 simulated scenarios. These scenarios were based on a combination of two batch sizes, two types of batch allocation systems, four farm risk levels of BRD, and three treatment interventions.

### Batch sizes

We considered two possible batch sizes: small batches of 20 animals and large batches of 100 animals (both cases entailed 10 batches in total). We intended to assess whether the proposed tool is effective for various batch sizes, as there is considerable heterogeneity in batch sizes across the globe [21, 22].

### Batch allocation systems

We investigated two types of batch allocation systems (sorted by individual risk level and randomly allocated). In Random scenarios, the animals were randomly distributed among batches; thus, all batches differed in composition. In the Sorted scenarios, we consider that the farmer has access to prior risk level information and allocate animals into batches accordingly, grouping those of similar risk.

### Risk levels of BRD

Four scenarios on farm risk level proportions were assessed: Low, Medium, Balanced, and High. This categorization allowed the testing of contrasting scenarios reflective of real situations.

The scenarios were defined at the farm scale by the proportions of animals on the farm belonging to one of the three possible individual BRD risk levels (low, medium and high). Table 1 shows the proportions used in each of the four risk level scenarios.

**Table 1:** **Definitions of the four scenarios on farm risk level proportions**.

		Farm-wise proportion of individual risk level (%)		
		Low-risk	Medium-risk	High-risk
Scenarios	Low-risk level	90	10	0
	Medium-risk level	10	90	0
	High-risk level	0	10	90
	Balanced-risk level	30	40	30

For the Sorted scenarios, the animals were grouped into batches according to their individual risk level. Therefore, the same proportions were used but at the batch scale. For example, in the Low-risk level scenario, 90% of the 10 batches were composed exclusively of low-risk animals, and 10% of the batches were exclusively composed of medium-risk animals.

### Treatment interventions

We tested three treatment interventions: individual treatment of the detected animals, conventional collective treatment, and collective treatment triggered by early warnings (the proposed tool). The conventional collective treatment consists of using a threshold of 10% of the batch size. For the collective treatments triggered by early warnings, several thresholds were tested (further explained in the Supporting Information), being the best threshold 0.05. This means that when the estimated risk of infection was higher than 0.05, an alarm was triggered, and collective treatment was initiated.

### Hierarchical multivariate binomial dynamic generalized linear model

Dynamic generalized linear models (DGLMs) represent a specific type of state‒space model employed when monitoring non-normally distributed observations. The primary objective of a DGLM is to estimate an underlying parameter vector $\left( \theta \right)$ from observed data while considering the prior knowledge available before any observations are made [12].

It is not possible for farmers and veterinarians to accurately determine the true number of infected animals because of the existence of subclinical cases and the lack of perfectly reliable diagnostic tests. Therefore, we used a DGLM to estimate the true risk of infected animals with BRD and alert the farmer/veterinarian when the estimated risk of infection exceeds a predefined threshold, acting as an early warning system.

Risk is here defined as the probability of an arbitrary animal at risk being infected with BRD. To estimate the true underlying risk of BRD infection, we used four variables that can be observable by the farmers/veterinarians per batch $\left( j \right)$ and at each time step $\left( t \right)$. These variables included the number of animals considered by the farmer as infected, here called detected $\left( D \right)$; the number of hyperthermic animals $\left( H \right)$; the number of animals with mild clinical signs $\left( M \right)$; and the number of animals with severe clinical signs $\left( C \right)$. As multiple variables are considered simultaneously in the model, it is called a multivariate DGLM. Those variables were monitored as risks, such as the following:

$$R_{Xjt} = \frac{{y_{Xjt} }}{{N_{Xjt} }},$$

where $R_{Xjt}$ is a naive estimate for the risk of state $X \in \left\{ {D,{ }H,{ }M,{ }C} \right\}$ in batch $j$ at time step $t$, $y_{Xjt}$ is the number of new cases, and $N_{Xjt}$ is the population at risk.

The observations $y_{Xjt}$ of each state and batch at time $t$ were binomially distributed with probability $p_{Xjt}$ and number of trials $N_{Xjt}$ since they had two possible outcomes, such as being *detected* or *not detected*, *hyperthermic* or *not hyperthermic*, and so forth, making it a binomial DGLM. It is natural to model the risk on a logistic scale:

$$\eta_{Xjt} = {\text{log}}\left( {\frac{{p_{Xjt} }}{{1 - p_{Xjt} }}} \right),$$

where $\eta_{Xjt}$ corresponds to the logistic transformation of the risk for each state, batch and time step. In turn, the risk was equal to $\left( {\exp \left( { - \eta_{Xjt} } \right) + 1} \right)^{ - 1}$.

A DGLM consists of an observation equation and a system equation. These equations work synergistically to capture the underlying dynamics and relate them to observable measurements.

### Observation equation

The risks of being detected, hyperthermic, and having mild or severe clinical signs in each batch on a logistic scale ($\eta_{Xjt}$) were defined as follows:

$$\eta_{Xjt} = \mu_{1t} + \delta_{jt} + \beta_{X} ,$$

where $\mu_{1t}$ is the true risk of infection for batch 1 at time $t$; $\delta_{jt}$ is the *deviation* between the true risk for batch $j$ and batch 1 (accordingly, $\delta_{1t} = 0$ by definition); and $\beta_{X}$ is an observation bias for state $X$ reflecting that the observations of detections, hyperthermia and clinical signs (mild and severe) were indirect observations of the true infection state. Thus, they were assumed to be biased.

At each time step, four observation pairs $\left( {y_{{Djt{ }}} ,N_{Djt} } \right),{ }\left( {y_{{Hjt{ }}} ,N_{Hjt} } \right),{ }\left( {y_{{Mjt{ }}} ,N_{Mjt} } \right),{ }\left( {y_{{Cjt{ }}} ,N_{Cjt} } \right)$ were performed for each of the $J = 10$ batches. Then, we define

$$\eta_{t} = \left( {\eta_{D1t} , \eta_{H1t} , \eta_{M1t} ,\eta_{C1t} ,\eta_{D2t} , \eta_{H2t} , \eta_{M2t} ,\eta_{C2t} , \ldots , \eta_{DJt} , \eta_{HJt} , \eta_{MJt} ,\eta_{CJt} } \right)^{\prime }$$

as a vector of logistically transformed binomial probability parameters that depend on an underlying parameter vector ($\theta_{t}$), as described by the following *observation equation*:

$$\eta_{t} = F_{t}^{\prime } \theta_{t}$$

where $\theta_{t} = \left( {\mu_{1t} ,{ }\delta_{2t} ,\delta_{3t} ,\delta_{4t} ,\delta_{5t} ,\delta_{6t} ,\delta_{7t} ,\delta_{8t} ,\delta_{9t} ,\delta_{Jt} ,{ }\beta_{D} ,\beta_{H} ,\beta_{M} ,\beta_{C} } \right)^{\prime }$ and where $F_{t}^{\prime }$ is a design matrix linking the parameter vector to the transformed binomial probabilities. This matrix represents how the risks of infection in each batch were related to the risk of each of the four states and their respective biases at each time step. The number of rows in $F_{t}^{\prime }$ corresponds to the four states in each batch $\left( {4 \times 10} \right)$, and the number of columns corresponds to the size of $\theta_{t}$; thus, it is a $40 \times 14$ matrix:

$$F_{t}^{\prime } = \left[ {\begin{array}{*{20}c} 1 & \cdots & 0 & \cdots & 0 & 1 & 0 & 0 & 0 \\ 1 & \cdots & 0 & \cdots & 0 & 0 & 1 & 0 & 0 \\ 1 & \cdots & 0 & \cdots & 0 & 0 & 0 & 1 & 0 \\ 1 & \cdots & 0 & \cdots & 0 & 0 & 0 & 0 & 1 \\ \vdots & \ddots & \vdots & \ddots & \vdots & \vdots & \vdots & \vdots & \vdots \\ 1 & \cdots & 1 & \cdots & 0 & 1 & 0 & 0 & 0 \\ 1 & \cdots & 1 & \cdots & 0 & 0 & 1 & 0 & 0 \\ 1 & \cdots & 1 & \cdots & 0 & 0 & 0 & 1 & 0 \\ 1 & \cdots & 1 & \cdots & 0 & 0 & 0 & 0 & 1 \\ \vdots & \ddots & \vdots & \ddots & \vdots & \vdots & \vdots & \vdots & \vdots \\ 1 & \cdots & 0 & \cdots & 1 & 1 & 0 & 0 & 0 \\ 1 & \cdots & 0 & \cdots & 1 & 0 & 1 & 0 & 0 \\ 1 & \cdots & 0 & \cdots & 1 & 0 & 0 & 1 & 0 \\ 1 & \cdots & 0 & \cdots & 1 & 0 & 0 & 0 & 1 \\ \end{array} } \right],$$

where the first column corresponds to the risk of infection of batch 1; the second column corresponds to the risk of infection of batch $j$; the third column corresponds to the risk of infection of batch 10; and columns 4–7 correspond to the biases of each of the four states $D$, $H$, $M$ and $C$ (in this order). The first four rows represent the risk of infection of $D$, $H$, $M$ and $C$ (in this order) for batch 1; rows 5–8 correspond to the risk of infection of $D$, $H$, $M$ and $C$ for batch $j$; and rows 9–12 correspond to the risk of infection of $D$, $H$, $M$ and $C$ for batch 10. Our approach consisted of using batch 1 as the reference, meaning that all the remaining batches had their risks presented as deviations from batch 1, thereby creating a hierarchical DGLM.

### System equation

The *system equation* expresses the evolution of the parameter vector over time. The general form of the system equation is as follows:

$$\theta_{t} = G_{t} \theta_{t - 1} + w_{t} , \quad w_{t} \sim \left[ {\underline {0} , W_{t} } \right],$$

where $G_{t}$ represents the system matrix. Since no patterns or trends were expected, $G_{t}$ was assumed to be equal to the identity matrix. The notation $w_{t} \sim \left[ {\underline {0} , W_{t} } \right]$ means that $w_{t}$ (random error) has zero mean $(\underline {0}$ is a vector of zeros) and a known variance‒covariance matrix $W_{t}$, which describes how much the risk of infection of each batch and the bias of each state are likely to randomly change over time. The systematic variance‒covariance matrix was assumed to be constant so that $W_{t} = W$, and its dimensions correspond to the size of $\theta_{t}$, being an $14 \times 14$ matrix.

We created two structured $W$ matrices. For the scenarios where the animals were randomly allocated into batches (Random scheme), we created a specific variance‒covariance matrix $\left( {W_{R} } \right)$, where we used one parameter considering the herd level variance $\left( {\sigma_{h}^{2} } \right)$ and another parameter for the batch level variance $\left( {\sigma_{b}^{2} } \right)$:

$$W_{R} = \left[ {\begin{array}{*{20}c} {\sigma_{h}^{2} + \sigma_{b}^{2} } & \cdots & {\sigma_{h}^{2} } & \cdots & {\sigma_{h}^{2} } & 0 & 0 & 0 & 0 \\ \vdots & \ddots & \vdots & \ddots & \vdots & \vdots & \vdots & \vdots & \vdots \\ {\sigma_{h}^{2} } & \cdots & {\sigma_{h}^{2} + \sigma_{b}^{2} } & \cdots & {\sigma_{h}^{2} } & 0 & 0 & 0 & 0 \\ \vdots & \ddots & \vdots & \ddots & \vdots & \vdots & \vdots & \vdots & \vdots \\ {\sigma_{h}^{2} } & \cdots & {\sigma_{h}^{2} } & \cdots & {\sigma_{h}^{2} + \sigma_{b}^{2} } & 0 & 0 & 0 & 0 \\ 0 & \cdots & 0 & \cdots & 0 & 0 & 0 & 0 & 0 \\ 0 & \cdots & 0 & \cdots & 0 & 0 & 0 & 0 & 0 \\ 0 & \cdots & 0 & \cdots & 0 & 0 & 0 & 0 & 0 \\ 0 & \cdots & 0 & \cdots & 0 & 0 & 0 & 0 & 0 \\ \end{array} } \right],$$

so that the variances and covariances of the risks of infection of all batches changed according to $\sigma_{h}^{2}$, since they all belonged to the same herd. For the variances, we added the batch effect $\sigma_{b}^{2}$. The values for the biases were set as 0 because the biases were not expected to change over time.

For the scenarios where the animals were sorted into batches by risk level (Sorted scheme), we used a different variance‒covariance matrix $\left( {W_{S} } \right)$. It consists of the same structure as $W_{R}$ with one extra parameter considering the risk level $\left( {\sigma_{r}^{2} } \right)$. We added $\sigma_{r}^{2}$ to the variances of the risks of infection of all batches and to the covariances of the batches with the same risk level. If we consider that the batches represented in columns 1 and 2 have the same risk level (and differ from the batch represented by column 3), their covariances also change according to $\sigma_{r}^{2}$:

$$W_{S} = \left[ {\begin{array}{*{20}c} {\sigma_{h}^{2} + \sigma_{b}^{2} + \sigma_{r}^{2} } & \cdots & {\sigma_{h}^{2} + \sigma_{r}^{2} } & \cdots & {\sigma_{h}^{2} } & 0 & 0 & 0 & 0 \\ \vdots & \ddots & \vdots & \ddots & \vdots & \vdots & \vdots & \vdots & \vdots \\ {\sigma_{h}^{2} + \sigma_{r}^{2} } & \cdots & {\sigma_{h}^{2} + \sigma_{b}^{2} + \sigma_{r}^{2} } & \cdots & {\sigma_{h}^{2} } & 0 & 0 & 0 & 0 \\ \vdots & \ddots & \vdots & \ddots & \vdots & \vdots & \vdots & \vdots & \vdots \\ {\sigma_{h}^{2} } & \cdots & {\sigma_{h}^{2} } & \cdots & {\sigma_{h}^{2} + \sigma_{b}^{2} + \sigma_{r}^{2} } & 0 & 0 & 0 & 0 \\ 0 & \cdots & 0 & \cdots & 0 & 0 & 0 & 0 & 0 \\ 0 & \cdots & 0 & \cdots & 0 & 0 & 0 & 0 & 0 \\ 0 & \cdots & 0 & \cdots & 0 & 0 & 0 & 0 & 0 \\ 0 & \cdots & 0 & \cdots & 0 & 0 & 0 & 0 & 0 \\ \end{array} } \right].$$

The values for the biases were also set as 0.

The parameters used for $W_{R}$ $(\sigma_{h}^{2}$ and $\sigma_{b}^{2} )$ and for $W_{S}$ $(\sigma_{h}^{2} , \sigma_{b}^{2}$ and $\user2{ }\sigma_{r}^{2} )$ were estimated by numerical optimization as the values that minimized the root mean square error (RMSE) of the forecast errors. We used two learning sets with a Balanced risk level for estimating the parameters in $W_{R}$ and $W_{S}$, one with a Random scheme and the other with a Sorted scheme. The built-in function *optim* in R [23] was used for the estimation, with the optimization algorithm specified as Nelder‒Mead. The estimated parameters used in this study can be found in the Supporting Information.

### Initialization

To fully specify the DGLM, we need to provide the matrices ${ }F_{t}$, ${ }G_{t} ,$ $W_{R}$ and $W_{S}$ along with the initial distribution of ${ }(\theta_{0} |D_{0} ) \sim N \left( {m_{0} , C_{0} } \right)$*.* The initial belief at time $0$ is represented by ${ }D_{0}$, which consists of an initial mean $\left( {m_{0} } \right)$ with the same dimension as $\theta_{t}$, and a variance‒covariance matrix ${ }(C_{0} )$, which has a $14 \times 14$ structure.

Both the initial $m_{0}$ and $C_{0}$ were first arbitrarily defined. For the initial ${ }m_{0}$, the risks of infection of all batches were defined as the logistic transformation of $0.01$ (the risks of infection of batches 2 to 10 were given as deviations from batch 1) and as $0$ for the biases; thus, $m_{0} = \left( { - 4.56, 0, \ldots ,0} \right)$. For the initial $C_{0}$, we set the variances for the risks of infection of all batches as $4$ and for the biases equal to ${ }8$. Afterwards, to find the best possible estimates of $m_{0}$ and $C_{0}$, we utilized the previously created $m_{0}$ and $C_{0}$ to run a retrospective analysis called smoothing [12]. To achieve this, we used a learning set exclusively composed of low-risk individuals with 20 animals per batch (small batch size).

Some adjustments were made to the smoothed $m_{0}$ and $C_{0}$ for the Medium, Balanced, and High-risk scenarios since they were created with a low-risk level dataset. In the Random scheme, the herd risk level was known, but the allocation of animals into batches was randomly performed. Therefore, for the initialization process, we considered that all the batches had the same risk level (equal to the herd risk level). On the other hand, for the Sorted scheme, the risk level of each batch was known. Therefore, each batch was addressed according to its risk of infection. In both cases, the risks of infection in $m_{0}$ and the variances and covariances for the risks of infection in $C_{0}$ were increased for the batches that were defined as having Medium or Balanced-risk and even further increased for the High-risk batches. The magnitude of these increases and the methodology employed can be found in the Supporting Information.

The biases in $m_{0}$ and $C_{0}$ were kept the same as those given by the smoothed $m_{0}$ and $C_{0}$, as we did not expect them to change between scenarios since they were farmer dependent. However, the covariances in $C_{0} { }$ between the biases and the risks of infection of the Medium, Balanced, and High-risk batches had to be adjusted. The reader is referred to the Supporting Information for further explanation.

### Sequential update

The theoretical foundation of a univariate binomial DGLM is well established in the literature [12]. However, for our specific study, it became necessary to extend this framework to a multivariate binomial DGLM. Therefore, to update the model at each time step, a Taylor expansion of the probability density function of $y_{t}$ given $\eta_{t}$ was employed, as described in Appendix A of Bono et al. [13].

In some cases, the rank of ${ }F_{t}^{\prime }$ was less than the number of rows. This led to the formation of a singular variance‒covariance matrix, which cannot be inverted; thus, sequential updating was not possible. In such cases, a stepwise updating technique was also employed, as described in Appendix A of Bono et al. [13].

### Global pipeline workflow

**Figure 1:**
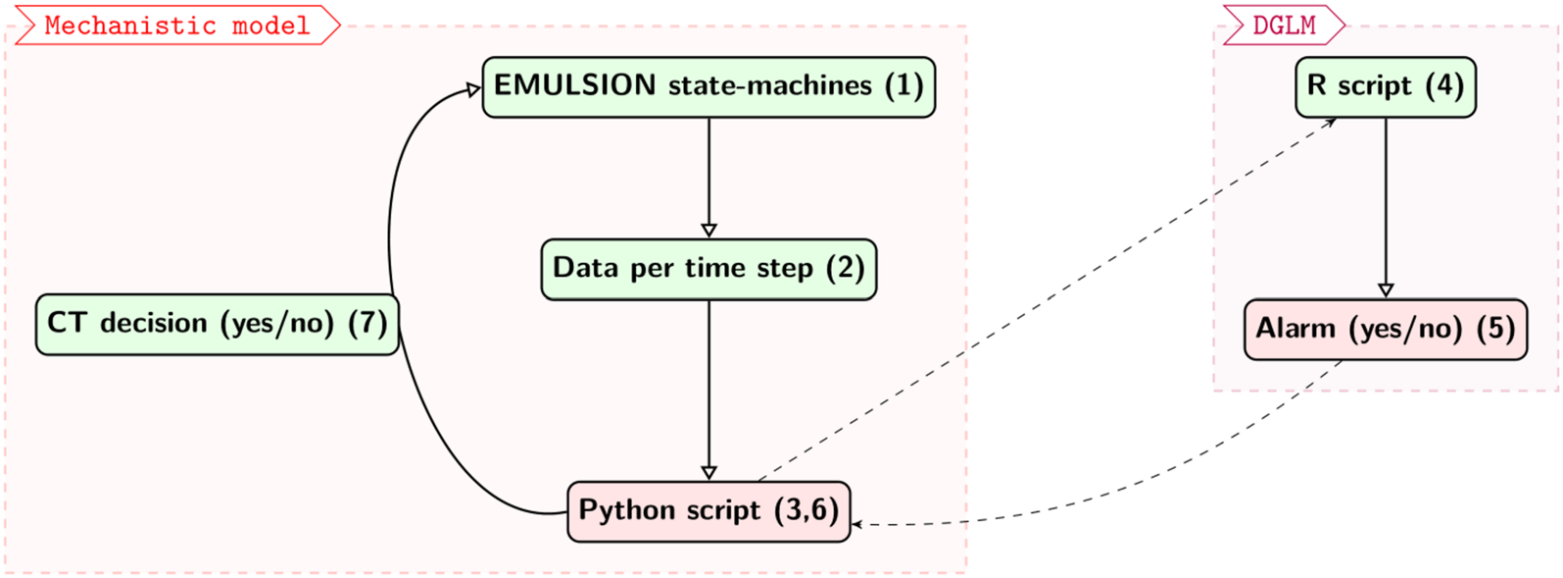
**Global pipeline workflow**. The EMULSION state machines (1) generate synthetic data at a given time step (2). These data go through a python script (3) that runs the execution of the DGLM by the R script (4) with the data (2) given as input. The DGLM script outputs an alarm file (5) that contains a Boolean per batch, determining if an alarm was triggered in any given batch at the current time step. This alarm file is returned to the Python script (6), which completes the loop to the state machines with a collective treatment decision at the current time step (7)

The next step consisted of connecting the mechanistic model with the DGLM. The global pipeline workflow is presented in Figure 1. The interface between the mechanistic model and the DGLM was established in Python. At each time step, the EMULSION state-machines (1) create synthetic data for the specific time step and save it in a CSV file (2). The data were sent to a Python script (3), which called the execution of the DGLM (4). In the R script, the DGLM analyses the data and estimates the risk of infection for each batch. If the estimated risk of infection exceeded the chosen threshold (0.05; see the Supporting Information), the DGLM triggered an alarm. The R script subsequently wrote a CSV file containing booleans determining if an alarm was triggered by the DGLM in any batch (5). Python adds-on retrieved these booleans (6) and sends that information back to EMULSION state machines (7). If the decision was *yes* in at least one batch, EMULSION triggered collective treatment for the specific batch. The pipeline continued to work as explained above for all the time steps. A collective treatment was carried out at most once in each batch.

### Outputs

For each scenario, 200 stochastic replicates were performed. Each stochastic replicate simulated the first 40 days after entering a fattening farm featuring 10 batches. The following outputs were compared across scenarios:

- Cumulative incidence: For each stochastic replicate of each scenario, we summed the number of new cases over the course of the simulation. We then computed the median of this quantity.
- Average time for severe clinical signs: For each stochastic replicate of each scenario, we summed the time spent by each animal in the state of severe clinical signs. We divided this by the cumulative incidence to obtain the average time spent with severe clinical signs per affected animal. We then computed the median of this quantity.
- Antimicrobial usage (AMU): The number of doses used in each stochastic replicate of each scenario was counted and divided by the population size. The median of this quantity was computed.
- Risks of infection: The estimated risk of infection by the DGLM was recorded for each stochastic replicate of each scenario where the collective treatments were based on early warnings. The empirical risk of infection at time $t$ was also computed as the ratio between the true number of newly infected individuals at time $t \left( {I_{t} - I_{t - 1} } \right)$ and the number of individuals at risk of being infected at $t - 1$. The individuals at risk consisted of susceptible animals $\left( S \right)$ and asymptomatic carriers $\left( E \right)$. This translated to $\varrho_{t} = \frac{{I_{t} - I_{t - 1} }}{{S_{t - 1} + E_{t - 1} }}$. For the Random scenarios, the means were computed according to the scenarios. For the Sorted scenarios, the means were computed by batch category *(i.e.,* batches composed only of low-, medium-, and high-risks).

## Results

### Cumulative incidence

**Figure 2:**
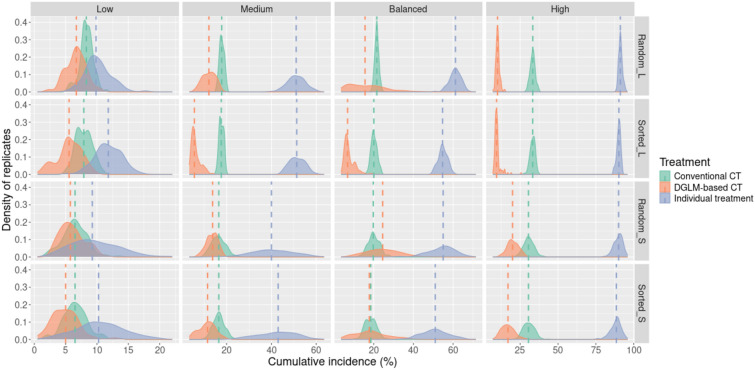
**Distribution of the cumulative incidence across the 48 scenarios**. The dashed lines represent the median of the distributions. L-large; S-small; CT-collective treatment

Compared with conventional collective treatment, collective treatment triggered by early warnings yielded a lower median cumulative incidence in all cases, except for the Balanced-risk scenarios with small batches (Figure 2). This treatment strategy performed especially well in the High-risk scenarios and with larger batches. Moreover, sorting batches seemed to further reduce the incidence.

### Average time to severe clinical signs

**Figure 3:**
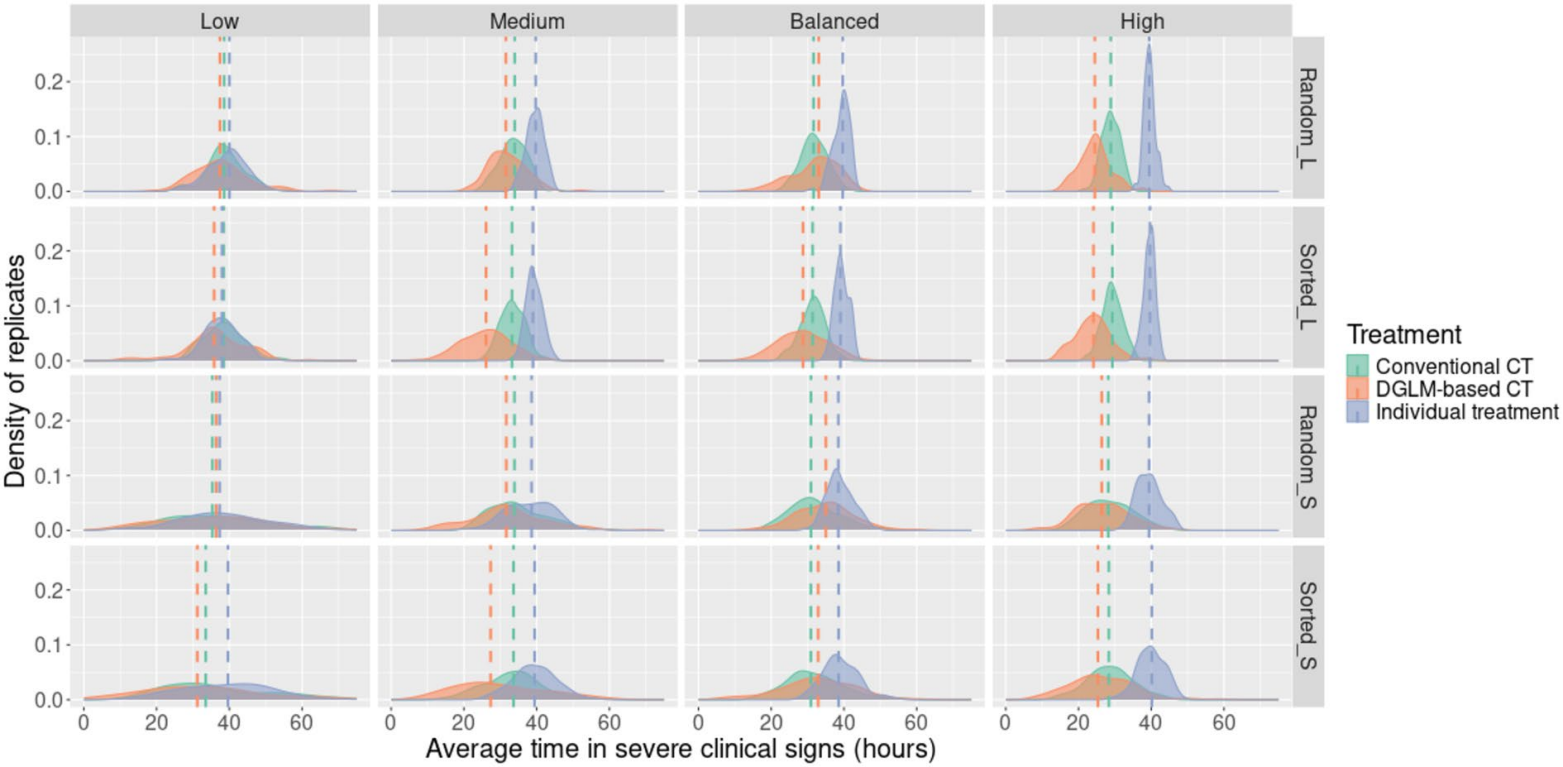
**Average duration of severe clinical signs (in hours) for an infected animal across the 48 scenarios**. The dashed lines represent the median of the distributions. L-large; S-small; CT-collective treatment

DGLM-based collective treatments yielded a lower median average time for severe clinical signs than their conventional counterparts in the Medium and High-risk scenarios (Figure 3: 2^nd^ and 4^th^ columns). Moreover, sorting batches seemed to further reduce the severity in Medium-risk scenarios. In the Balanced-risk scenarios, conventional collective treatment strategies resulted in slightly shorter durations of severe clinical signs, except for larger, sorted batches. In the Low-risk scenario, there was no clear difference in the duration of severe clinical signs among the three treatment strategies, except in the small, sorted batches, where the scenario with collective treatment based on the DGLM yielded the shortest duration of severe clinical signs.

### AMU

**Figure 4:**
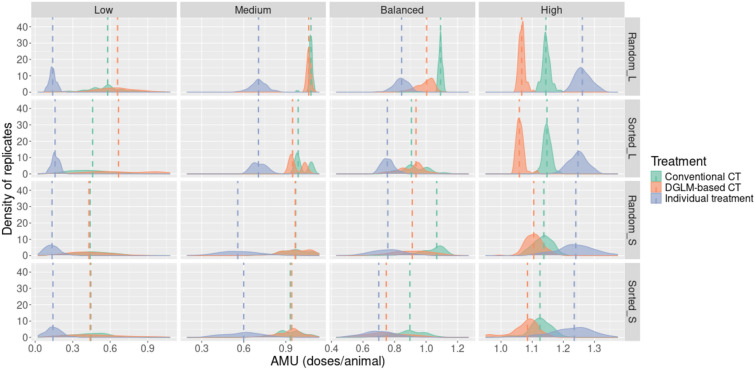
**Distributions of AMU across the 48 scenarios**. The dashed lines represent the median of the distributions. L-large; S-small; CT-collective treatment

The collective treatment strategy based on early warnings was associated with lower AMU than the other two strategies in High-risk scenarios, especially in large batches (Figure 4). In Balanced and Medium-risk scenarios, the DGLM-based collective treatments yielded lower or equal AMU than did the conventional collective treatment, except for the large sorted Balanced-risk scenario, where the collective treatments based on early warnings had a slightly higher AMU. Moreover, in Low-risk scenarios with larger batches, AMU was the highest for collective treatment triggered by early warnings. In the Low, Medium and Balanced-risk scenarios, both collective treatment strategies were associated with higher AMU than individual treatments.

We observed that the density of the AMU distributions could display local maxima (Figure 4). In the Medium-risk scenarios, we could observe up to 3 local maxima at regular intervals on the x-axis of the density plots. For the Low-risk scenarios, the density appeared to be wider, with less clear peaks.

### Risks of infection

**Figure 5:**
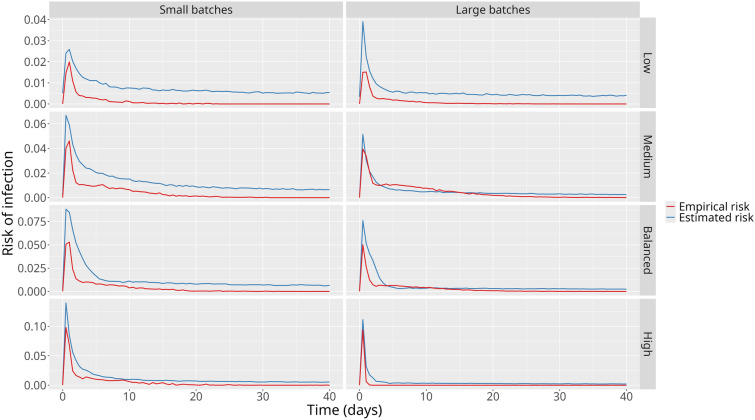
**Mean empirical and estimated risks of infection in the Random scenarios**

Among scenarios where animals were randomly allocated to batches, scenarios with larger batches presented a smaller difference between the estimated and empirical risks of infection (Figure 5). The scenarios with a majority of low-risk animals presented the greatest difference. On the other hand, the smallest difference was found in the Medium and High-risk scenarios. Overall, there was a consistent overestimation of the estimated risk of infection.

**Figure 6:**
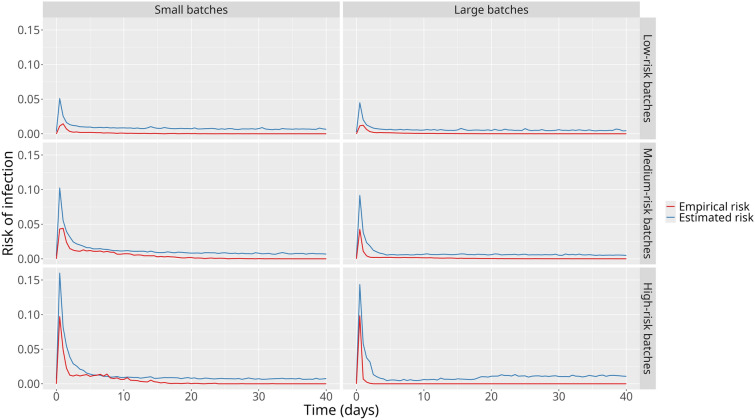
**Mean empirical and estimated risks of infection in the Sorted scenarios**

In the sorted scenarios, the estimated risk of infection had the smallest difference from the empirical risk in batches featuring high-risk animals, especially in larger batches (Figure 6). Conversely, batches featuring only low-risk individuals presented the greatest difference. Similar to the Random scenarios, an overestimation of the risk of infection was also observed.

## Discussion

As a proof of concept, we demonstrated that a decision support tool combining automated early warnings and a mechanistic epidemiological BRD model in a multibatch cattle fattening farm helps better trigger collective treatments to both limit AMU and BRD cases, as well as identify situations where individual treatments should be preferred over collective ones. We compared 48 scenarios in terms of cumulative incidence, average time spent with severe clinical signs, and AMU. Our findings suggest that the use of DGLM-based collective treatments is highly beneficial in High-risk scenarios and is considered advantageous in Medium and Balanced-risk scenarios. Despite some overestimation, the risk of infection estimated by the DGLM had a strong association with the empirical risk, emphasizing the ability of the DGLM to adequately predict the trends of the risk of infection in batches using data on clinical signs. Nevertheless, the DGLM consistently overestimated the risk without necessarily affecting the outputs. Our findings illustrate the potential of this tool in enabling the timely implementation of collective treatment interventions, providing valuable guidance for improving animal welfare and AMU.

We showed that DGLM-based collective treatments could efficiently reduce the incidence of bacterial respiratory disease in populations with a high individual risk of BRD. This reduction is especially important in larger batches. This finding is consistent with a previous simulation study that revealed that larger High-risk batches tended to benefit more from collective treatments [16], but such treatments were triggered using the conventional approach. The American system often implements prophylactic treatments for at-risk cattle at the time of feedlot entry [24]. In our study, a metaphylactic treatment strategy based on early warnings was associated with a reduction in the cumulative incidence of BRD cases in large batches, which is similar to the farming context in America and Australia [24]. These results were acquired without preventive treatment upon arrival. Future studies could compare the performance of collective treatment strategies based on early warnings and preventive treatment upon arrival.

Our study also demonstrated that the average duration of severe clinical signs was reduced by the implementation of DGLM-based collective treatments in herds mainly composed of high- and medium-risk individuals. This reduction could be due to a shorter delay between the onset of clinical signs and treatment, which assessed the timely response of the collective treatment based on early warnings. Reducing the duration of severe clinical signs is crucial for animal welfare. Furthermore, clinical signs are associated with reduced daily weight gain, which causes economic losses to farmers [25]. Thus, the timely response generated by early warnings could have beneficial welfare and economic impacts on the field.

Our simulations showcased the trade-off between antimicrobial consumption and the reduction of BRD incidence and severity. While the benefit of performing collective treatments for High-risk scenarios is clear in this study, in the remaining scenarios, collective treatments successfully reduced the incidence and severity, but they were associated with a larger AMU than the baseline individual treatment. This compromise is advantageous in Medium and Balanced-risk scenarios, where the reduction in incidence is substantial. While this trade-off was already reported by Picault et al. [16], our study showed that, compared with conventional collective treatments, DGLM-based collective treatments have the advantages of reducing AMU, incidence and severity, leading to a more advantageous trade-off.

Our results showed that the individual risk level proportions strongly influence the performance of the treatment strategies. Indeed, collective treatment strategies were globally relevant for incidence and severity reduction in Medium, Balanced, and High-risk scenarios, but they did not present an advantage in Low-risk situations. These findings suggest that characterizing the overall stress and risk of respiratory diseases could be highly beneficial and could help in decision-making regarding treatment strategies. The preconditioning programs put in place in US farming systems frequently characterize animal risk levels to process high-risk cattle separately or to perform prophylactic treatment upon arrival. These programs have been studied in Europe, but it is suggested that the sector should focus on beef sector reorganization and other husbandry practices, as the results of preconditioning programs were inconclusive [26].

Sorting the animals according to their risk level into batches further enhanced the benefits of the DGLM-mediated response. As shown by Sorin-Dupont et al. [15], such batch allocations usually perform well in reducing the circulation of bovine respiratory pathogens. In our study, it further reduced disease occurrence in all situations and disease severity in Medium-risk scenarios when DGLM-based collective treatments were implemented. This suggests that combining the DGLM with transparent information on stress and husbandry practices would allow better farm management and the implementation of suitable treatment strategies.

In some scenarios with collective treatment, we observed multiple local maxima in the density function of the AMU across the replicates. This could be due to collective treatments being triggered at the batch level. Therefore, each local maximum could correspond to a given number of batches having triggered collective treatment in a particular scenario. We noticed the absence of a local maximum for the High-risk scenario, suggesting that collective treatments were probably triggered in every batch. The difference observed in terms of cumulative incidence and average duration of severe clinical signs could thus have been due to the early nature of the response. Therefore, these results indicated that the DGLM-mediated response was timelier than the conventional response.

We estimated the risk of infection of each batch at each time step based on information about clinical signs and the number of animals that were detected as infected. Then, the estimated risks of infection were compared with the true risks of infection (empirical) used by EMULSION to generate the synthetic data. The DGLM correctly anticipated the peak of the risk of being infected, although the estimated peak is higher than the empirical one, and the estimated risk does not decay as fast as the empirical risk. This led to an overestimation. From an operational point of view, this did not affect the outputs in scenarios with higher risk, as the alarms were triggered in a timely manner. The subsequent overestimation by the DGLM had no impact afterwards, as collective treatments could be triggered only once. This overestimation did, however, lead to higher AMU in Low-risk scenarios, as it sometimes triggered unnecessary collective treatment. This situation could be avoided by using a less sensitive threshold for low-risk batches, provided that information on individual risk levels is available. Another possible solution might be to develop a multiprocess DGLM that distinguishes between different states: the normal situation (absence of BRD), outliers, and level shifts (presence of BRD). This approach has been applied to other contexts; for example, a multiprocess model was used to detect when sows were in heat [27]. Modelling these states separately might reduce the overestimation issue by better adapting to changes in infection risk. This approach should be explored in future work.

In parallel, it is also relevant to consider how the model-based approach used compares to simpler alternatives. A less technically demanding alternative to the use of the DGLM would be to use the conventional collective treatment strategy with an updated intervention threshold. While this might be a simpler possibility, relying on threshold tuning considering the number of detected animals is insufficient. The key limitation of such an approach is that it assumes that the number of animals detected by the farmer or veterinarian as infected with BRD accurately reflects the true number of infected animals. In reality, this is unlikely due to the presence of subclinical cases, the imperfect sensitivity and specificity of available diagnostic tests, and the fact that detection accuracy often depends on the individual expertise and judgment of farmers and veterinarians. In contrast, the DGLM aims to estimate the underlying true risk of infection by using data on the observed clinical signs and by accounting for uncertainty. As a result, the DGLM offers a more reliable and epidemiologically meaningful basis for intervention decisions.

This novel tool combines mechanistic modelling with automated early warnings based on the DGLM estimated risk of infection. To the best of our knowledge, no other combinations of these two modelling techniques exist in animal health. Using mechanistic modelling allowed us to generate synthetic data for various scenarios, which we then used to test the relevance of the response by the early warnings. We also used it to evaluate treatment interventions in terms of cumulative incidence, disease severity and antimicrobial usage. An interesting approach is to adapt this tool to run on observed on-farm data in real time so that farmers and veterinarians can make more informed treatment decisions, with customized guidance on how to manage BRD cases. Indeed, the DGLM part of the tool could trigger an alarm based on these real data and provide an estimate of the risk of infection for each batch of the farm at the time of the alarm. This alarm being provided to the EMULSION BRD model, the tool could eventually forecast what may happen if the veterinarian decides to perform a collective treatment in response to the alarm and compare it to what may occur if the conventional threshold for collective treatment is used or if only individual treatments are performed. Within the tool, farmers can input the number of animals per batch and the number of batches on their farm since the proposed tool can work with different values than the ones used in this study.

This study provides a basis for building a broad decision-making tool for ranking treatment strategies regarding BRD. Future work should also include other respiratory pathogens and the cocirculation of different pathogens, as well as other livestock species.

## Conclusion

The present study successfully demonstrated the potential of integrating mechanistic epidemiological modelling with an automated early warning system to optimize decision-making regarding collective treatments for BRD. By simulating various scenarios using synthetic data, we found that collective treatments triggered by early warnings provided the best overall outcomes for Medium, Balanced, and High-risk scenarios. In Low-risk scenarios, individual treatments performed better, suggesting the need for tailored strategies based on BRD risk levels. The importance of this study lies in its innovative approach, which combines two modelling techniques to create a novel decision support tool. This tool has the potential to improve animal welfare by reducing both BRD cases and the duration of severe clinical signs while also addressing the critical issue of antimicrobial resistance through reducing antimicrobial usage. Further validation with real-world, on-farm data is necessary. If implemented in real time, this tool could offer veterinarians and farmers more informed and customized guidance for managing BRD cases.

## Supplementary Information

**Additional file 1** **Supporting Information**. Graphic overview of the mechanistic model simulating the spread of *M. haemolytica*, explanation of the processes of the mechanistic model, parameters used for the systematic variance‒covariance matrices, DGLM initialization methodology, and threshold selection for the DGLM-based collective treatments.).

## Data Availability

Access to the codes utilized in developing the models detailed in this study is publicly available at 10.5281/zenodo.13842980.
