# Supplementary material for: Combining dynamic generalized linear models and mechanistic modelling to optimize treatment strategies against bovine respiratory disease
Source: Vet Res. 2025 Sep 25;56:181. doi: 10.1186/s13567-025-01611-y (PMC12465693; doi:10.1186/s13567-025-01611-y)
Supplement: Supplementary file 1 — Additional file 1 Supporting Information. Graphic overview of the mechanistic model simulating the spread of M. haemolytica, explanation of the processes of the mechanistic model, parameters used for the systematic variance‒covariance matrices, DGLM initialization methodology, and threshold selection for the DGLM-based collective treatments.). [file 13567_2025_1611_MOESM1_ESM.pdf]

Supporting information

Contents

1 SI1: Model graphic overview **1**
1.1 Model overview for *M.haemolytica* 1

2 SI2: Model processes **2**
2.1 Processes of the model 2
2.2 Hyperthermia 3
2.3 Infection and clinical signs status 3
2.4 Detection status 3
2.5 Treatment status 3

3 SI3: Parameters used for the systematic variance-covariance matrices for the Random (W_R) and for the Sorted (W_S) scenarios **4**

4 SI4: Initialization methodology **4**

5 SI5: Threshold selection for the DGLM-based collective treatment **5**
5.1 Method 5
5.2 Results 5

1 SI1: Model graphic overview

This supplementary information provides graphic overview of the states and processes of the model. We propose a formalism featuring the transitions from one state to another as well as the regulatory links existing between transitions. In this formalism, full arrows represent a transition from one state to another whereas the dashed lines represent regulatory links. Let X be a regulator (state or transition) of transition T_2. This will be represented by a dashed arrow starting from X and targeting T_2.

1.1 Model overview for *M.haemolytica*

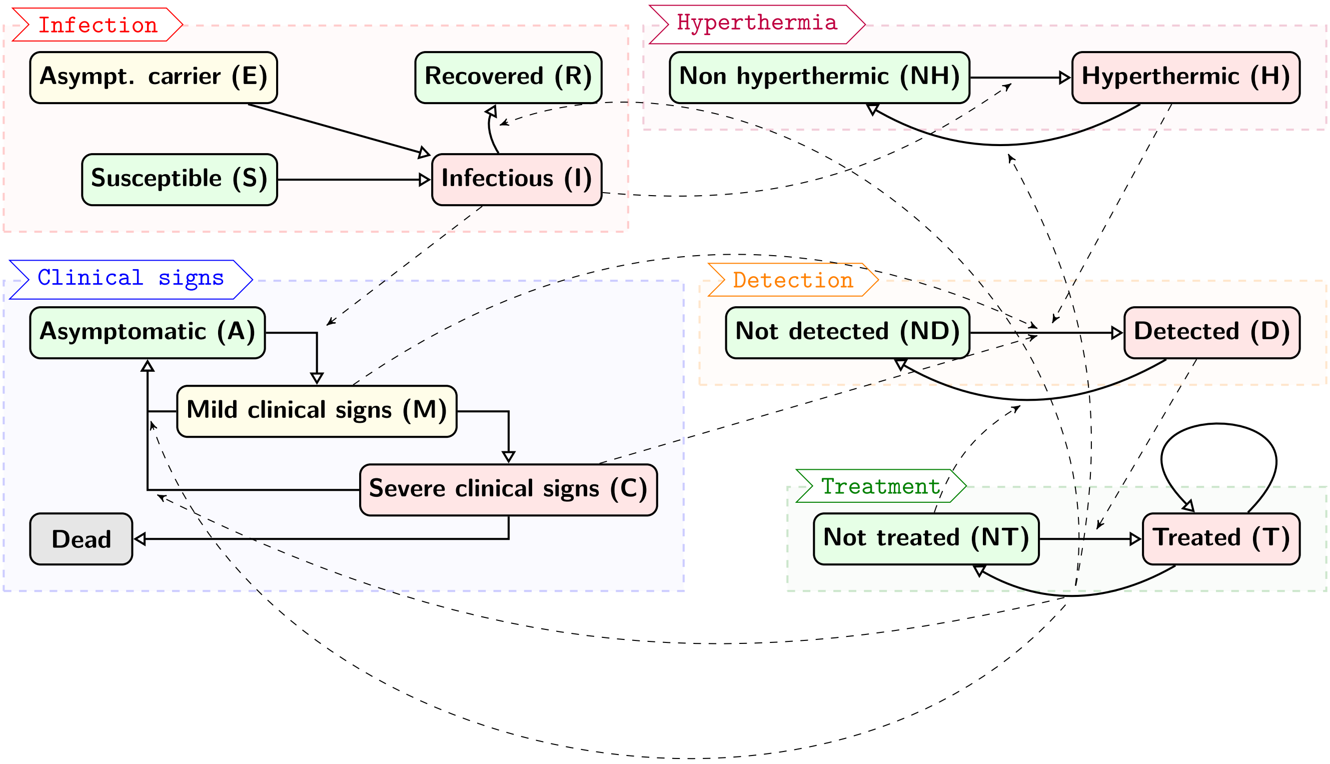

Figure S1: Graphic overview of the states and transitions in each process of the model. Transitions are represented by full arrows, while dashed lines represent regulatory links.

2 SI2: Model processes

2.1 Processes of the model

In the model, five processes drive the states of individuals: infection, hyperthermia, clinical signs, detection and treatment. They are represented by a formalism broadly used in computer science, finite state machines, which is close to flow diagrams used by epidemiologists, with a higher expressiveness. Their textual description in the YAML format in the model file (link to the model repository) is easy to read and can be automatically converted in a graphical representation by EMULSION. To help understand the state machine diagrams, we present how the diagram summarising the infection process relates to the state machine specification, extracted from the whole model. More details on how state machines differ from flow diagrams are available in the documentation: https://sourcesup.renater.fr/www/emulsion-public/pages/Modelling_principles.html. The following subsections are directly drawn from Sorin-Dupont et al. (2023) [15] as they describe processes and transitions that remained unchanged from this previous version of the model.

2.2 Hyperthermia

Hyperthermia was composed of two states: hyperthermic (H) and non-hyperthermic (NH). NH animals could transition to H with probability p_H attributed to non-infectious factors. Once in state H, they remained there for a period τ_H sampled from a Beta distribution adjusted using observed data, before reverting to NH. Additionally, the transitions from NH to H and back could result from the infection process.

2.3 Infection and clinical signs status

Four health statuses were considered: susceptible (S), asymptomatic carrier (E), infectious (I) and resistant (R) animals. Asymptomatic carriers could spontaneously turn I with probability p_E and could also be infected by surrounding infectious individuals (I). Three actions were triggered when entering the state I: (1) the individual exhibited mild clinical signs for a duration τ_M drawn from a Beta distribution calibrated from observed data, (2) the animal changed from NH to H state, (3) a random draw with probability p_C drove whether the individual would display severe clinical signs at the end of its mild clinical signs. If displaying severe clinical signs, a boolean deciding on the survival of the individual was drawn from a binomial law of probability p_d. Death occurred at the end of the severe clinical signs duration (τ_C). If the individual did not die from infection, it then recovered and became resistant (R). Recovery occurred after duration τ_I drawn from a gamma distribution according to the given bacterial pathogen. Theoretically, τ_I is longer than τ_M. However, $\tau_M + \tau_C$ could exceed τ_I. In that case, the infectious period was $\tau_M + \tau_C$. When transitioning to R state, animals changed from H to NH.

2.4 Detection status

Two detection methods were used. The first detection relied on visual on-farm appraisal of clinical signs, assuming lethargy was the most significant sign to calibrate the delay (τ_M) between infection and severe sign occurrence. Severe clinical signs were detected with a sensitivity of 1, while the sensitivity for mild clinical signs detection was assumed to be 0.5. The model assumed a clinical check-up at every time step (12 hours). Following the detection of the first case through visual appraisal, all hyperthermic animals were identified and transitioned frome undetected state to detected using rectal temperature measured at the next feeding time, 12 hours later.

2.5 Treatment status

Each animal detected as diseased or hyperthermic, or in the context of collective treatment transitioned from not treated (NT) to treated (T). Treated animals received one antibiotic dose, assumed to be effective after a certain duration τ_T. If animals still exhibited clinical signs after this duration, they would be treated again for the same duration, but the number of treatments per individual per episode was limited (max_T). Transitions from T to NT occurred in three cases: (1) recovery after τ_T due to successful treatment with probability p_T, (2) the end of the infectious period occurred while under treatment but was not caused by it, (3) treatment failure after max_T doses. A treatment success triggered the transition from I to R, consequently trigerring transitions driven by the end of the infectious state *i.e.* the transitions to non-hyperthermic and to asymptomatic states.

3 SI3: Parameters used for the systematic variance-covariance matrices for the Random (W_R) and for the Sorted (W_S) scenarios

The parameters used for W_R (σ_h^2 and σ_b^2) and for W_S (σ_h^2, σ_b^2 and σ_r^2) were estimated using the built-in function `optim` in R with the optimization algorithm specified as Nelder-Mead. After running the algorithm, the estimated parameters for W_R were $\sigma_h^2 = 0.02784$ and $\sigma_b^2 = 0.10143$, and for W_S were $\sigma_h^2 = 0.20254$, $\sigma_b^2 = 0.04299$ and $\sigma_r^2 = 0.11257$.

4 SI4: Initialization methodology

The initial mean (m_0) and the variance-covariance matrix (C_0) were created using a learning set exclusively composed by low-risk level individuals. Therefore, for the Medium, Balanced, and High-risk scenarios some adjustments in m_0 and C_0 were necessary. In the Random scheme, as the allocation of animals into batches was done randomly, we assumed that all the batches had the same risk level, equal to the herd risk-level. For the Balanced scenarios we considered that all the batches had a medium-risk level. In the Random case, the risks of infection in m_0 were increased by 1.5 across all batches on the Medium and Balanced scenarios, and by 3 on the High-risk scenarios. The variances and covariances for the risks of infection in C_0 were also increased since we are less sure about the true values of m_0 on those cases than on the Low-risk scenarios. We increased the variances and covariances for the risks of infection in C_0 by 2.2 and $\sqrt{2.2}$ respectively across all batches on the Medium and Balanced scenarios, and by 2.5 and $\sqrt{2.5}$ on the High-risk scenarios.

For the Sorted scheme, the risk level of each batch was known. Therefore, the risks of infection in m_0 and the variances and covariances for the risks of infection in C_0 were increased only for the batches composed by medium and high-risk animals. Similarly to the Random scheme, the risks of infection in m_0 were increased by 1.5 on the batches featuring medium-risk animals and by 3 on batches with high-risk animals. The variances and covariances for the risks of infection in C_0 were also increased, but to a smaller extent compared to the Random scheme. In the Sorted case, we were more certain about the true values of m_0 because we knew the risk level of each batch and we took that into account when creating m_0. Consequently, the variances and covariances for the risks of infection in C_0 were increased by 1.2 and $\sqrt{1.2}$ respectively on the batches with medium-risk animals, and by 1.5 and $\sqrt{1.5}$ on batches featuring high-risk individuals. The covariances of the risks of infection in C_0 between batches with different risk levels were adjusted by multiplying the square root of the coefficients used to increase the variances of the two batch risk levels being considered (for low-risk batches we used 1).

The biases in m_0 and C_0 were kept the same. This decision was based on the assumption that these biases, being farmer-dependent, were unlikely to undergo changes across different scenarios. However, the covariances in C_0 between the biases and the risks of infection had to be adjusted. For the Random scheme, we multiplied the covariances in C_0 between the biases and the risks of infection by $\sqrt{2.2}$, and $\sqrt{2.5}$ for the medium and high-risk batches, respectively. For the Sorted scheme, we multiplied by 1, $\sqrt{1.2}$, and $\sqrt{1.5}$ for the low, medium and high-risk batches, respectively.

5 SI5: Threshold selection for the DGLM-based collective treatment

5.1 Method

In scenarios featuring collective treatment triggered by alarms generated by the Dynamic Generalized Linear Model (DGLM), the alarms were triggered when the risk of infection estimated by the DGLM went above a certain threshold. Such a threshold thus had to be defined as a preliminary step of our study.
In order to determine the relevant threshold for intervention, we defined a range of viable threshold values. As shown in Figure 5 and Figure 6 in the main text, the estimated risk of infection seldom goes above 0.3. We thus took $]0, 0.3]$ as our range of plausible thresholds. We then discretized this range by steps of 0.05. The model was then run 50 times on every scenario featuring DGLM-based collective treatment with each threshold value.
We then compared the cumulative incidence and antimicrobial use (AMU) for each threshold value in each scenario in order to select the threshold value minimizing the cumulative incidence while not overusing antimaticrobials.

5.2 Results

The cumulative incidence was the lowest for a threshold value of 0.05 in every scenario (Figure [S2]).

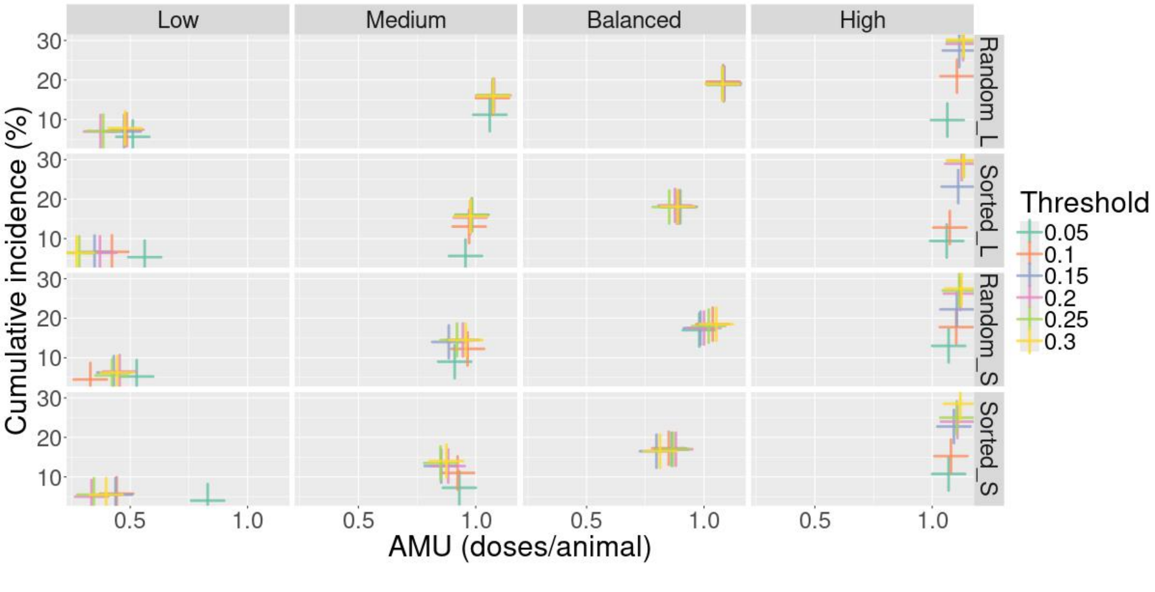

Figure S2: Median cumulative incidence and antimicrobial use (AMU) across scenarios and thresholds for DGLM based intervention. Columns: risk level proportion. Rows: batch allocation. Low: 90, 10 and 0% of low, medium and high-risk individuals respectively. Medium: 10, 90 and 0% respectively. High: 0, 10 and 90% respectively. Balanced: 30, 40 and 30% respectively. Random: the animals are randomly assigned into batches. Sorted: the animals are assigned into batches according to their individual risk level. L: batches of 100 animals each; S: batches of 20 animals each.

As this value was the lowest among the tested interval, we tested another interval,]0,0.1], with steps of 0.01 and followed the same operations as described in the Method section. The results showed that a threshold of 0.05 was the best trade-off between a reasonable AMU in Low-risk scenarios and a proper reduction of the incidence in Medium and High risks (Figure S3). Overall, this threshold value was not associated with higher AMU than the other values.

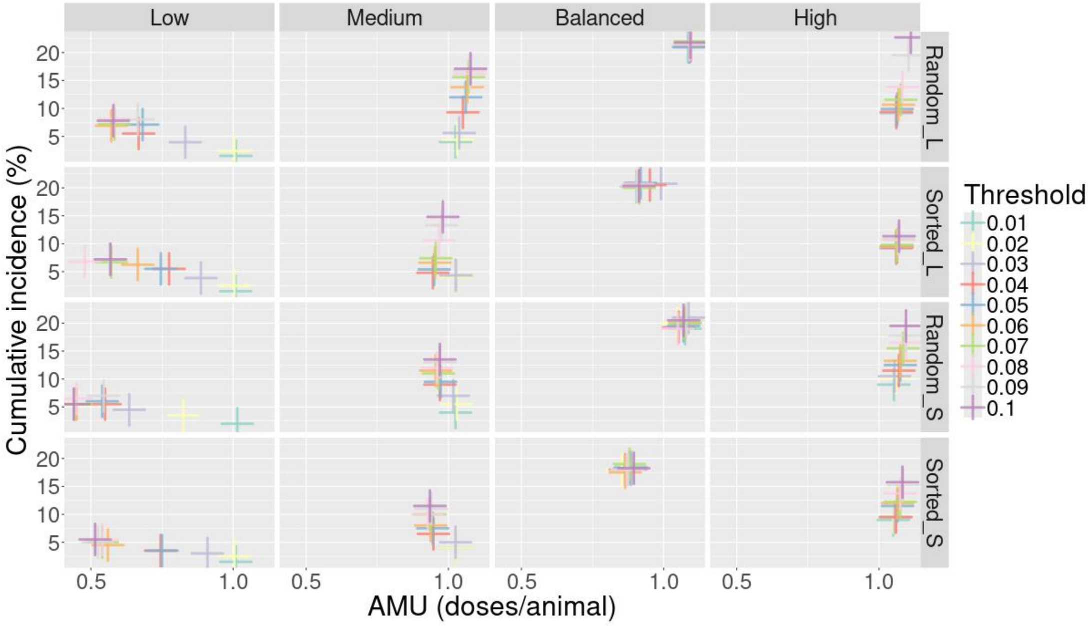

Figure S3: Median cumulative incidence and AMU across scenarios and thresholds for DGLM based intervention. Each column represents a risk level proportion and each row represent a batch allocation.

We privileged having a balance between an overall reduction of the cumulative incidence while having a reasonable AMU. **We thus use 0.05 as the threshold on estimated risk of infection triggering an alarm.**
